# Review: The HSP90 molecular chaperone—an enigmatic ATPase

**DOI:** 10.1002/bip.22835

**Published:** 2016-05-20

**Authors:** Laurence H. Pearl

**Affiliations:** ^1^ Genome Damage and Stability Centre, School of Life SciencesUniversity of SussexFalmerBrightonBN1 9QRUK

**Keywords:** molecular chaperones, conformational change, ATP, client protein, cochaperone

## Abstract

The HSP90 molecular chaperone is involved in the activation and cellular stabilization of a range of ‘client’ proteins, of which oncogenic protein kinases and nuclear steroid hormone receptors are of particular biomedical significance. Work over the last two decades has revealed a conformational cycle critical to the biological function of HSP90, coupled to an inherent ATPase activity that is regulated and manipulated by many of the co‐chaperones proteins with which it collaborates. Pharmacological inhibition of HSP90 ATPase activity results in degradation of client proteins in vivo, and is a promising target for development of new cancer therapeutics. Despite this, the actual function that HSP90s conformationally‐coupled ATPase activity provides in its biological role as a molecular chaperone remains obscure. © 2016 Wiley Periodicals, Inc. Biopolymers 105: 594–607, 2016.

## INTRODUCTION

The HSP90s are a family of molecular chaperones that function in the cellular stabilization, regulation, and activation of a range of ‘client’ proteins.^1^, ^2^ One or more cytosolic HSP90 isoform is found in all eukaryotes, and many higher eukaryotes also possess specialized mitochondrial (TRAP1) and endoplasmic reticulum (GRP94/GP96/endoplasmin) isoforms.^3^, ^4^ An HSP90 homolog, HtpG, is also found in eubacteria, but although its deletion generates mild phenotypes,^5^ clear roles are yet to emerge. Remarkably HSP90/HtpG homologs are totally absent from the archaea.^6^

The clientele of eukaryotic cytosolic HSP90s covers a broad spectrum of protein classes and structural types, within which the eukaryotic protein kinases, and transcription factors such as steroid hormone receptors, form the largest coherent groups.^7^ HSP90 is also implicated in the assembly of RNA polymerases, PI3‐kinase‐like kinases such as mTOR and SMG1, snoRNPs^8^ and NLR innate immunity receptors,^9^ amongst others. Recent high‐throughput protein–protein interaction screens^10^ have also identified a large number of E3 ubiquitin ligase subunits as at least interacting with HSP90, but whether these are actually dependent as a class on HSP90 for their function remains to be shown.

**Figure 1:**
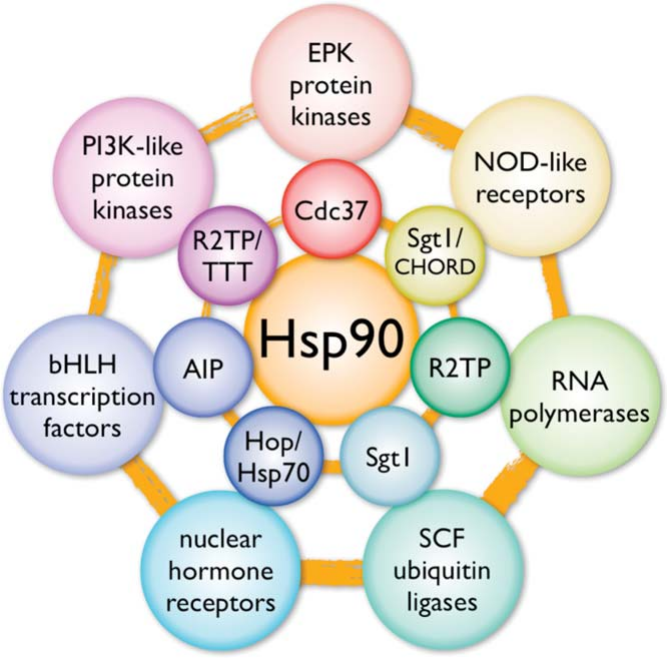
HSP90 and its clients. HSP90 engages with a range of ‘client’ protein classes (outer circle) via its interaction with various co‐chaperone proteins or complexes (inner circle), that act as adaptors, simultaneously able to interact with the specific client and the central chaperone itself.

The ability of HSP90 to engage with its plethora of client proteins is mediated by a set of co‐chaperones that act as adaptors and encapsulate the specificity and selectivity for client proteins.^1^, ^11^ The best understood, and in some ways the simplest of these adaptor co‐chaperones is Cdc37 (a.k.a. p50), which mediates recruitment of members of the eukaryotic Ser/Thr and Tyrosine kinase family^12^ and forms a stable ternary complex with HSP90 and the kinase client that has been amenable to structural analysis.^13^ Steroid hormone receptors such as the oestrogen and glucocorticoid receptors are recruited to HSP90 via preliminary interaction with a member of the Hsp70 family of molecular chaperones, coupled to HSP90 via the TPR‐domain protein Hop/Sti1.^14^, ^15^ Other TPR‐domain co‐chaperones such as AIP and FKBP38 have been implicated in recruitment of such diverse proteins as aryl hydrocarbon receptor^16^ and hepatitis C NS5A^17^ to the HSP90 system, but the mechanistic details of these processes are not yet well understood. The most complex HSP90 co‐chaperone system described to date, is the R2TP (RuvBL1/2‐Tah1/RPAP3‐Pih1) complex^18^ – a complex of four different proteins recruited to HSP90 via the TPR‐domain component Tah1p (yeast) or RPAP3/Spagh1 (metazoan).^19^, ^20^ R2TP couples HSP90 to stabilization and assembly of snoRNPs, RNA Pol II,^21^ and via its onwards interaction with the Tel2‐Tti1‐Tti2 complex, the PI3 kinase‐like kinases, SMG1, and TOR.^22^ (Figure 1)

## CONTROVERSY OVER THE ATP DEPENDENCE OF HSP90

Early studies of the HSP70 and CPN60/GroEL classes of molecular chaperones had revealed a clear functional dependence on the binding and hydrolysis of ATP.^23^, ^24^ This generated much speculation that the HSP90 chaperones would share this property. ATPase activities were indeed detected in HSP90 preparations purified from bacteria, trypanosomes, yeast, and human cells^25^, ^26^ but the apparent affinity for ATP and the kinetic parameters of its turnover varied widely. An inherent autophosphorylating protein kinase activity and ATP‐dependent conformational changes of HSP90 were also reported.^27^, ^28^ However subsequent biochemical and biophysical studies of highly purified recombinant yeast^29^ and human^30^ HSP90s appeared to show conclusively that, unlike HSP70, HSP90 neither bound nor hydrolyzed ATP. These studies suggested that the highly variable ATPase activities previously observed were because of contaminating ATPases or protein kinases, and supported a previous model of HSP90 as a passive chaperone with affinity for non‐native conformations of unfolded proteins.^31^

This issue was ultimately resolved by a crystallographic study of yeast HSP90, which unambiguously identified a highly conserved binding site for ATP within the N‐terminal domain of the chaperone.^32^ The ATP‐binding site in the N‐terminal domain is structurally quite distinct from ATP‐binding sites in other chaperones, but is distantly related to those of prokaryotic DNA Gyrase and eukaryotic type II topoisomerases, the MutL DNA mismatch repair proteins, and the histidine kinases of prokaryotic two‐component signaling systems, together with which it forms the ‘GHKL’ family of ATPases.^33^, ^34^

## THE HSP90 ATP‐BINDING SITE

**Figure 2:**
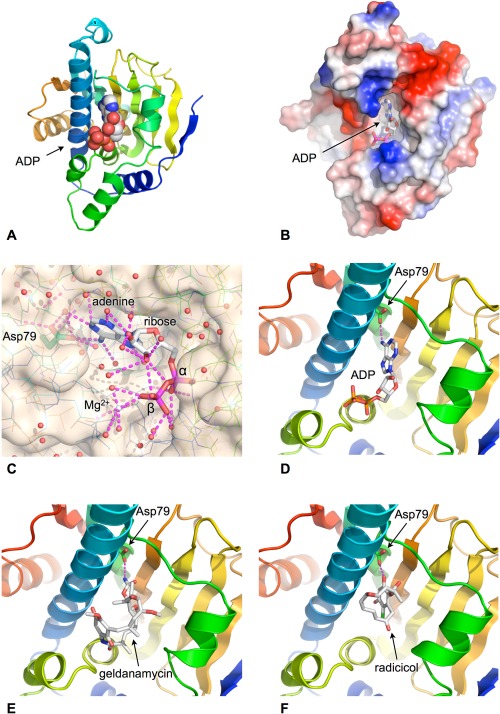
ATP‐binding site. A: ATP/ADP binds into a pocket formed in the helical face of the N‐terminal domain. Cartoon shows secondary structural elements rainbow colored according to reative position within the primary structure of the domain – blue: N‐terminus, red: C‐terminus. B: Molecular surface of HSP90 N‐domain colored according to electrostatic potential blue:+ve, red:‐ve. The adenine base is buried in the binding pocket with the phosphates exposed to solvent. C: Close up of the water‐filled nucleotide‐binding pocket. Polar interactions made by the bound ADP are shown as dashed lines. D: The adenine base makes only a single direct hydrogen bond with the protein, via the side chain of Asp 79. E: The natural product geldanamycin binds to the nucleotide‐binding pocket and is a competitive inhibitor of ATP binding by HSP90. F: The natural product radicicol binds to the nucleotide‐binding pocket and is a competitive inhibitor of ATP binding by HSP90.

The ATP‐binding site in HSP90 lies in a deep pocket on the helical face of the N‐domain, lined by predominantly hydrophobic residues^32^ (Figures 2A and 2B). In the crystal structure the bound nucleotide makes extensive interactions with the residues lining the pocket, and with a number of highly ordered solvent molecules that mediate many of the key polar interactions. The adenine base of the ATP penetrates deep into the pocket, with one face packed against hydrophobic side chains. However it only makes a single direct hydrogen bond to the side chain of Asp79; all its other polar interactions are water mediated. Bound water molecules also mediate most of the interactions with the ribose sugar, while the α and β phosphates of the ATP are coupled to the protein via an octahedrally coordinated Mg^2+^ ion (Figure 2C).

The ATP‐binding site revealed by the crystal structure overlaps with the binding sites for two different classes of microbial antibiotics, geldanamycin, and radicicol,^35^, ^36^ both of which had been previously shown to possess anti‐tumour activity and an ability to disrupt protein kinase signaling pathways,^37^, ^38^ although the mechanism of that was not fully understood (Figures 2D–2F). These compounds function as competitive inhibitors of ATP‐binding to HSP90, and allowed the inherent ATPase activity of the chaperone to be measured for the first time without interference from other contaminating activities.^39^

Yeast HSP90 in isolation displays a geldanamycin‐sensitive ATPase activity with a turnover of ∼0.2 min^−1^,^39^ considerably smaller than the rate of ∼150 min^−1^ previously reported^25^. A variety of assay systems yield *K*_m_ values in the range ∼500 μM for yeast and human HSP90s^40^, ^41^, ^42^ – a considerably higher value than the *K*_m_ ∼ 1 μM measured for HSP70 using comparable methods. This probably explains the considerable variability in early studies where the ATP concentrations used may not have fully saturated the chaperone.

The discovery and structural characterization of the ATP‐binding site in the N‐terminal domain of HSP90, made it possible to determine the degree to which the ATPase activity of HSP90 contributed to the essential biological functions of HSP90 as a molecular chaperone *in vivo*. Using engineered yeast strains, mutations were introduced into the N‐terminal domain of residues that were implicated by the crystal structure in binding or hydrolysis of ATP.^39^, ^43^ These mutations had dramatic effects on the viability of yeast, in which the HSP90 chaperone is essential, demonstrating that both binding and hydrolysis of ATP are essential to the *in vivo* function of HSP90, and confirming HSP90 unambiguously as an ATP‐dependent system.

## THERAPEUTIC TARGETING OF HSP90S ATPase ACTIVITY

**Figure 3:**
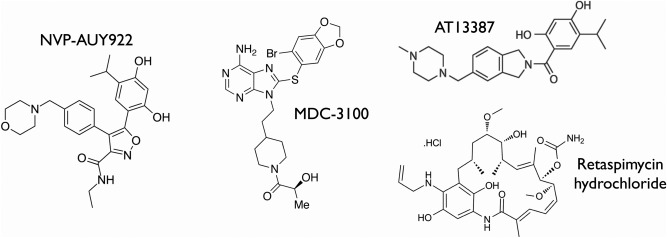
HSP90 inhibitors. Chemical structures of a number of synthetic or semisynthetic ATP‐competitive HSP90 inhibitors currently in clinical development.

The essential involvement of HSP90 in the stabilization of many highly oncogenic protein kinase clients^12^ and the revelation of HSP90 as an ATP‐dependent protein with an eminently ‘druggable’ nucleotide‐binding site in its N‐terminal domain, prompted a substantial level of interest in HSP90 as a therapeutic target in cancer.^44^, ^45^ Progress was greatly assisted by the availability of natural product ‘tool’ compounds such as geldanamycin and radicicol whose mode of action and binding sites were well characterized.^35^, ^36^ The validity of the approach was clearly demonstrated in early studies using modified versions of geldanamycin, such as 17AAG, which showed downregulation of key oncogenic client proteins such as ERBB2, CRAF, and AKT, and loss of signaling through critical cancer‐driving signaling pathways in cell lines and in patients.^46^, ^47^ The development of novel chemotypes not based on the natural products soon followed,^48^, ^49^ and there are now more than a dozen HSP90 ATP‐competitive inhibitors in clinical trial at various stages,^50^ with promising activity against a range of tumours^51^ (Figure 3).

## ATPase COUPLED CONFORMATIONAL CYCLE

**Figure 4:**
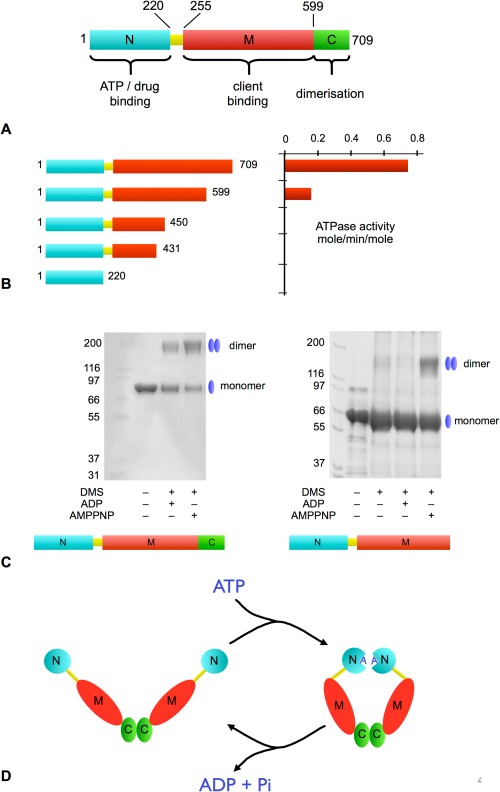
ATPase coupled molecular clamp mechanism. A: Domain architecture of HSP90. B: ATPase activity of HSP90 C‐terminal truncation mutants. There is a dramatic loss of activity following removal of the C‐terminal dimerization domain. C: Full‐length inherently dimeric HSP90 (left) can be chemically cross‐linked (DMS) regardless of whether it has ADP or an ATP analog (AMPPNP) bound. The C‐domain deleted HSP90 is monomeric when apo or bound to ADP, but is able to dimerize in the presence of AMPPNP demonstrating the presence of a second nucleotide‐dependent dimerization site. D: Schematic of the ATPase‐coupled molecular clamp mechanism, in which ATP binding promotes N‐terminal association to form the active ‘tense’ catalytically active state, which then relaxes on hydrolysis of ATP.

The discovery that HSP90 function depends on the ability to bind and hydrolyze ATP immediately begs the question of how this process occurs at molecular level. Insight into this came from studies of truncation mutants of HSP90 that remove the C‐terminal domain that confers constitutive dimerization of HSP90^52^ (Figure 4A). These monomeric constructs displayed a much lower catalytic activity, with the isolated N‐terminal domain itself having no detectable ATPase activity despite providing the overwhelming majority of the affinity of HSP90 for ATP^39^ (Figure 4B). Cross‐linking studies of these weakly active and inherently monomeric C‐terminal truncation mutants, revealed an additional dimerization interface that was dependent on ATP binding, and shown to be mediated by ATP‐dependent dimerization of the N‐terminal nucleotide domains^53^ (Figure 4C). This study also implicated a ‘lid’ segment in the N‐terminal domain, delimited by two highly conserved clusters of glycine residues, as a mobile structural feature that might respond to ATP‐binding.

These studies provided the first glimpse into the ATPase coupled conformational cycle of HSP90 in which binding of ATP promotes association of the N‐terminal domains within a constitutive dimer mediated by the C‐terminal domain, forming a catalytically competent ‘tense’ state. ATP hydrolysis relaxes the dimer, allowing the N‐terminal domains to dissociate and exchange the reaction products for fresh ATP, to continue the cycle. While many further subtleties and mechanistic details of this process have been uncovered since it was first proposed (see Ref. 54, 55 for recent reviews relating to asymmetry), this ATP‐driven ‘molecular clamp’ remains the defining biochemical mechanism at the heart of HSP90 function (Figure 4D).

## STRUCTURAL BASIS OF THE ATPase‐COUPLED MOLECULAR CLAMP

**Figure 5:**
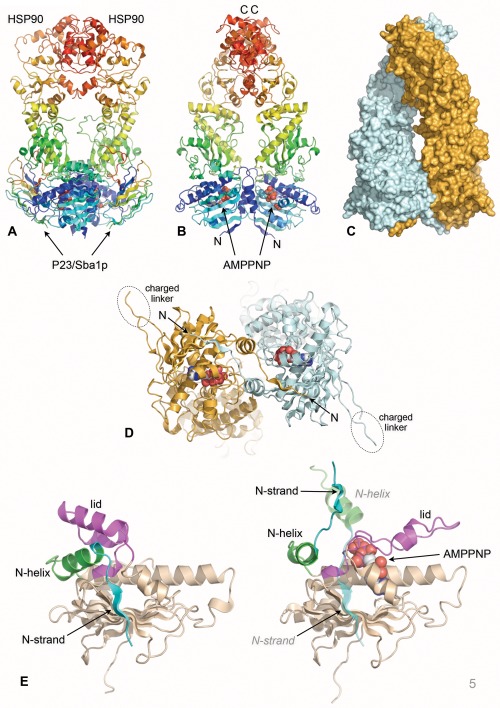
The ATP‐bound state. A: Secondary structure cartoon of the HSP90 dimer in the ATP‐bound closed state. The two protomers (rainbow coloured blue:N → red:C) make a constitutive dimer interaction at the C‐terminus and an ATP‐dependent dimer interface at the N‐terminus. The closed state is stabilized by binding of the co‐chaperone protein P23/Sba1 (see below). B: As (A), but rotated 45º around the vertical axis. The bound AMPPNP is shown as a CPK model. C: Space‐filling representation of the closed HSP90 dimer. The two protomers wrap around each other with a left‐handed twist. D: Close up of the dimerized N‐terminus showing the topological swap of the N‐terminal strand from each protomer. The visible parts of the highly flexible ‘charged linker’ that connects the N‐ and M‐domains is indicated. E: Binding of AMPPNP promotes a conformational change in key ‘switch’ regions in the N‐terminal domain.

Confirmation of the ‘molecular clamp’ model for the ATPase coupled conformational cycle of HSP90 was provided by the crystal structure of yeast HSP90 trapped in the predicted ‘tense’ conformation, bound to AMPPNP – a non‐hydrolysable ATP analog – and the co‐chaperone protein P23/Sba1 (see below)^56^ (Figure 5A). The structure reveals the HSP90 dimer in a highly compacted state, with the N‐terminal, middle and C‐terminal domains arranged in linear order along each of the protomers, which have a parallel arrangement with a left handed twist around the long axis of the dimer (Figures 5B and 5C).

As well as the constitutive dimer interface formed between the C‐terminal domains, a second intimate interface is formed between the two copies of the N‐terminal domain in the AMMPNP‐bound dimer, which undergoes considerable conformational rearrangement compared with its apo/ADP structure in isolation^32^ (Figure 5D). Restructuring of the N‐domain involves changes in two distinct ‘switch’ regions: (1) the N‐terminal β‐strand, which swaps over to hydrogen‐bond to the edge of the main β‐sheet in the N domain of the other protomer, with concomitant movement of the first α‐helix exposing a large hydrophobic patch that then forms a substantial dimer interface with the equivalent patch on the other protomer; and (2) the ‘lid’ segment, which swings through nearly 180º from its ‘open’ conformation in the isolated N‐domain, to fold over the nucleotide bound in the pocket and cradle the γ‐phosphate in a set of main‐chain hydrogen bonding interactions (Figure 5E).

**Figure 6:**
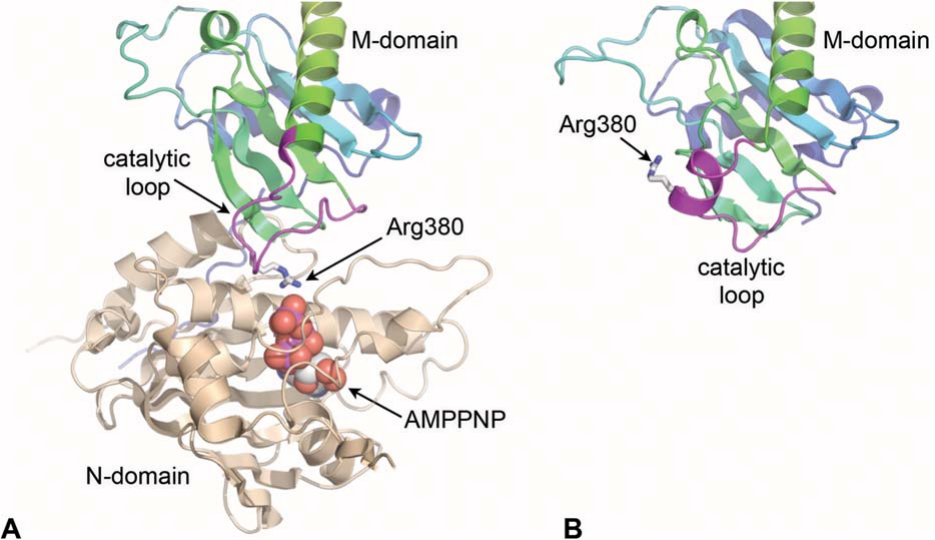
Formation of the ATPase active site. A: The conformational changes in the N‐terminal ‘switch’ regions that accompany binding of AMPPNP facilitate docking of the M‐domain and interaction of the conserved arginine residue with the γ‐phosphate of the nucleotide. B: Docking of the M‐domain is accompanied by substantial remodeling of the catalytic loop from its structure in the isolated M‐domain.

Dimeric association of the N‐domains is accompanied by docking of the structurally defined middle domain of Hsp90^57^ onto the N‐domain of the same protomer, bringing together residues from the lid segment in the N‐domain and a mobile loop from the middle (M) domain, cemented by an extensive hydrophobic interface. More limited intermolecular interactions also form between the N‐ and M‐domains of the individual protomers. The intramolecular N‐M interaction facilitates a conformational change in a third ‘switch’ region in the M‐domain, which brings a totally conserved arginine residue (Arg 380 in yeast) into contact with the γ‐phosphate of the ATP in the nucleotide‐binding pocket of the N‐domain, implicating it as a key part of the catalytic apparatus of the ATPase. Consistent with this, mutation of Arg380 had previously been shown to abolish ATPase activity *in vitro* and chaperone function *in vivo* 57 (Figure 6A). Subsequent studies have suggested that this arginine may not be directly involved in the chemical mechanism of ATP hydrolysis as such, but instead may act to stabilize the N‐M interaction in the presence of ATP^58^. Regardless of the precise role of Arg380, its engagement with the γ‐phosphate of ATP that accompanies docking of the M‐ and N‐domains, brings together the two halves of a ‘split’ active site that when assembled is able to catalyze the hydrolysis of the bound ATP.

More recently a structure of the closed state of the mitochondrial HSP90 TRAP1 has been obtained, using ADP and a number of transition‐state traps.^59^ All the dimeric interactions observed in the yeast HSP90 structure are present in the closed TRAP1 structure, confirming the universality of the model. A degree of asymmetry was observed between the two protomers in the TRAP1 structure compared with the yeast HSP90 structure, which has been taken to indicate that ATP‐hydrolysis in the two protomers may occur sequentially rather than simultaneously. This is consistent with biochemical observations that while both N‐domains in a dimer must be able to bind ATP for *in vivo* functionality, only one need be competent for ATP hydrolysis.^60^

The mechanistic model confirmed by this structure explains the functional effects of a number of HSP90 mutations described in the literature. An early genetic study in yeast identified a set of mutations that affected the ability of HSP90 to facilitate activation of client proteins.^61^ Two of the identified mutants Thr101Ile and Thr22Ile map to regions of the N‐terminus of HSP90 that undergo considerable changes in environment subsequent to ATP binding. Thr101 lies on the face of the lid segment that is packed against a hydrophobic patch on the N‐domain in the apo‐state, but becomes exposed on ATP‐binding. Mutation of this residue to the more hydrophobic isoleucine would be expected to stabilize the apo conformation of the lid, and indeed the Thr101Ile mutation decreases the ATPase activity to <10% of the wild type, with a concomitant decrease in the propensity of the N‐domains to associate.^53^ Thr22 maps to the end of the hydrophobic N‐terminal helix that associates with its equivalent on the other protomer on dimerization of the N‐domains. Consistent with its mutation to the more hydrophobic isoleucine, the Thr22Ile mutant enhances N‐domain association and substantially enhances the ATPase activity. That both of these mutants, which have very different effects on the ATPase activity of HSP90 *in vitro*, should generate comparable loss‐of‐function phenotypes *in vivo,* 61 highlights the importance of the rate of ATP turnover in HSP90s chaperone activity.

## A DEFINED OPEN STATE?

**Figure 7:**
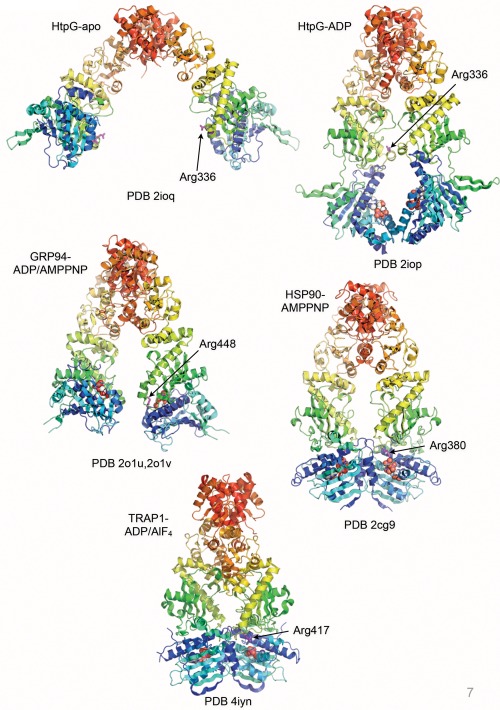
Conformational flexibility of HSP90. HSP90 family members display considerable conformational flexibility in the relative orientation of the C‐, M‐, and N‐domains within and between the protomers. Only in the case of the closed N‐terminally dimerized conformation seen in the crystal structures of yeast HSP90‐AMPPNP and TRAP1‐ADP/AlF_4_, is there any evident interaction that distinguishes ADP from ATP. PDB codes refer to the corresponding entries in the Protein Databank (PDB).

The structure of the ATP‐bound tense conformation unambiguously defines one pole of the ATPase‐coupled conformational cycle of HSP90, but there is far less clarity regarding the ‘relaxed’ state of the chaperone. Crystal structures of the yeast HSP90 dimer lacking the nucleotide‐binding N‐domain, the *E.coli* HSP90‐ HtpG in apo or ADP‐bound forms, and the ADP‐ or AMPPNP‐bound form of the mammalian endoplasmic reticulum HSP90 paralog GRP94,^56^, ^62^, ^63^ show a close and consistent juxtaposition of the C‐ and M‐ domains in each protomer, albeit with some flexing of the proximity of the two M‐domains to each other across the dimer. By contrast, there is tremendous variation in the orientation of the N‐terminal domain relative to the M‐domain in a range of HSP90, GRP94 and HtpG crystal structures^62^, ^63^, ^64^ (Figure 7). It has been suggested that the different N‐M orientations observed may reflect evolved functional differences amongst homologs and paralogs, and/or the existence of defined conformations fixed by the presence of ADP. However, it is very hard to eliminate the effects of the lattice in defining the juxtaposition of these very loosely coupled domains in the various crystal structures. Unlike the closed ‘tense’ state, there are no convincing mutational data that explain the significance of any of these conformations, or any biochemical explanation for how ADP, which has no effect on the various ‘switch’ segments, actually fixes the relative orientation of the N‐domain. It is highly likely that all these open conformations are largely conditioned by crystal contacts which in some cases are very substantial. The most extreme example of this is seen with the GRP94 structures,^63^ where the ADP‐ and AMPPNP‐bound proteins crystallize with the same unit cell and space group and are essentially identical.

By contrast, solution X‐ray scattering studies confirm a highly flexible conformational ensemble for the ‘relaxed’ state of HSP90,^65^, ^66^ so that the case for defined relative conformations of the N‐ and M‐domain other than the closed ATP‐bound state remains unproven.

## REGULATION OF THE ATPASE CYCLE BY HSP90 CO‐CHAPERONES

Early studies of HSP90 complexes *in vivo* identified a number of other proteins—so‐called co‐chaperones—that contribute to HSP90s biological function.^67^, ^68^, ^69^ Subsequent studies have shown that several of these achieve at least part of their function by regulating the ATPase activity of HSP90.^1^, ^70^

## HOP/Sti1p – HSP90/HSP70 COUPLING FACTOR

Regulation of HSP90s ATPase was first demonstrated for HOP/Sti1p (a.k.a P60)^71^ which couples the HSP90 and HSP70 chaperones as part of the chaperone‐mediated activation of steroid hormone receptors.^72^ HOP/Sti1p consists of an array of TPR domains that mediate its dimerization and interaction with the acidic peptide motif (‐EEVD‐COO^‐^) found at the C‐terminus of both of these chaperones.^73^, ^74^ Yeast Sti1p was found to form a stable dimer‐dimer interaction with HSP90 that completely blocked the ATPase activity of the chaperone.^71^ This property was not shared by other TPR‐domain co‐chaperones, although these could reactivate HSP90 by competitive displacement of Sti1p. Despite a number of subsequent biophysical and structural studies of both yeast and mammalian proteins,^75^, ^76^, ^77^ a clear consensus on the biochemical basis of this inhibitory activity is still lacking, although it most likely involves the stabilization of the HSP90 dimer in an open state in which N‐domain dimerization and N‐M domain docking is prevented, while client protein transfer from HSP70 is facilitated.^78^

## CDC37—PROTEIN KINASE RECRUITMENT ADAPTOR

**Figure 8:**
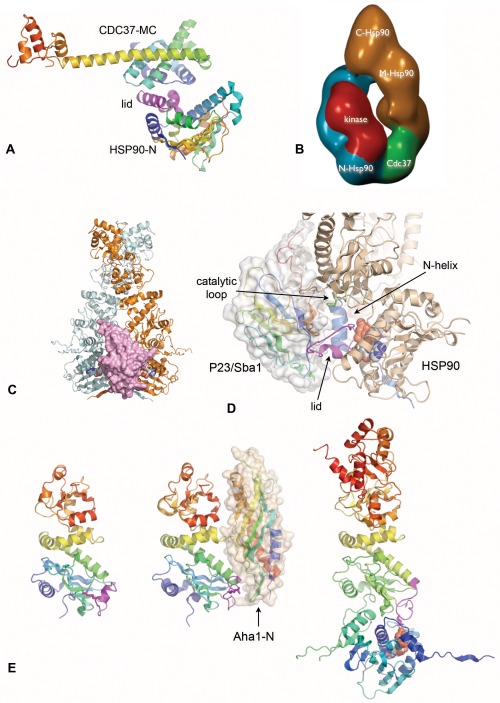
ATPase‐regulatory co‐chaperones. A: The middle and C‐terminal regions of CDC37 bind to the lid segment in the N‐domain of HSP90 and prevent its closure on ATP‐binding. B: Negative stain electron microscope single particle reconstruction of an HSP90‐CDC37‐CDK4 client protein complex. A single molecule of CDC37 and of the kinase bind asymmetrically to an HSP90 dimer. C: P23/Sba1 binds across the interface between the dimerized N‐terminal domains in the ATP‐bound closed state. D: Close up of P23/Sba1 complex with HSP90. The bound co‐chaperone contacts the three main switch segments – the lid (magenta), catalytic loop (green) and N‐terminal helix/strand (blue) but only in their ATP‐bound ‘tense’ conformations. E: The N‐terminal domain of Aha1 binds to the M‐domain of HSP90 (centre), and promotes the remodeling of the catalytic loop (magenta) from its inactive conformation as in the isolated M‐domain (left) toward the extended conformation seen in the ATP‐bound state (right) in which Arg380 contacts the γ‐phosphate of the ATP. The second HSP90 in the ATP‐bound dimer has been omitted for clarity.

The ability to arrest the ATPase cycle of HSP90 is shared by CDC37 (a.k.a. P50)^79^ ‐ the adaptor co‐chaperone required for the recruitment of protein kinase clients to the HSP90 system.^12^ CDC37 acts as a scaffold protein, with its N‐terminal region required for interaction with protein kinases, while its central and C‐terminal regions mediate interaction with HSP90. Structural studies show that the central helical domain of CDC37 binds to the lid‐segment in the HSP90 N‐domain, fixing it in the open conformation, and blocking the N‐domain dimerization and N‐M domain docking required for ATP hydrolysis^80^ (Figure 8A). CDC37 is itself a dimer and appears to interact with HSP90 in a dimer–dimer interaction that may fix the conformation of the chaperone in a way that facilitates client protein loading.^66^ However, in complexes with HSP90 and a client protein kinase, the CDC37 dimer is disrupted and a stable (HSP90)_2_ – CDC37 – kinase complex can be purified from cells^13^, ^81^ (Figure 8B). An additional binding site for CDC37 has been suggested in the middle domain of HSP90, but the biological significance of this interaction is yet to be determined.^82^

## P23/Sba1—A CONFORMATIONALLY SELECTIVE CO‐CHAPERONE

The P23 co‐chaperone was first identified as part of stable complexes of HSP90 with steroid hormone receptors.^83^ Association of P23 with the HSP90 system was found to be dependent on ATP,^84^ although the biochemical basis of this dependence was not understood at the time. The yeast homolog of P23, Sba1p^85^ shares this ATP‐dependency for binding to HSP90, and though unlike HSP90 it is not essential for yeast viability, HSP90 function *in vivo* is impaired in its absence.

Analysis of Sba1p binding to HSP90 mutants *in vitro* showed that Sba1p interaction depends not just on HSP90 binding ATP, but on its ability to respond to that ATP by undergoing a conformational change.^86^ This confirmed the idea of P23/Sba1p as recognizing a distinct conformational state of the HSP90 system, as had been suggested earlier.^84^

The biochemical basis for conformation‐dependent recruitment of Sba1p, was revealed by the crystal structure of yeast HSP90 bound to the non‐hydrolysable ATP analog AMPPNP, and Sba1p.^56^ The structure shows P23/Sba1p binding across the interface formed by the close association of the N‐domains of the HSP90 dimer in the ‘tense’ closed conformation (Figure 8C). Bound P23/Sba1p bridges between the N‐terminal α‐helix of HSP90 involved in formation of the hydrophobic interface between the dimerized N‐domains from one protomer, and the lid segment and M‐domain catalytic loop from the other protomer. P23/Sba1p binds to the surface of the HSP90 N‐domain lid that is usually buried in the apo structure, but is exposed when the lid closes over bound ATP. Similarly the interactions made by P23/Sba1p with the M‐domain catalytic loop are specific to the conformation observed when it is docked against the N‐domain with Arg380 extending across the N‐M interface to interact with the γ‐phosphate of ATP. Thus, P23/Sba1p binds specifically to all three of the main ‘switch’ segments in HSP90, but only in their closed ATP‐bound conformations (Figure 8D). However, once recruited, P23/Sba1p reinforces the ATP‐bound conformation of HSP90 and extends the lifetime of the closed state, potentially delaying release of ATP‐hydrolysis products. This is consistent with the observation that P23/Sba1p slows the ATPase cycle of HSP90 but cannot totally arrest it.^86^, ^87^

## Aha1—AN HSP90 ACTIVATOR

A genetic screen for high‐copy number suppressors of temperature‐sensitive yeast HSP90 mutants, identified a previously unknown protein – Hch1 – whose overexpression restored the function of the HSP90 mutant Glu381Lys.^88^ Although homologs of Hch1 were only identifiable in simple yeasts, Hch1 showed homology to the N‐terminal region of a second yeast protein Aha1, for which clear metazoan homologs could be identified. Biochemical analysis of Aha1 shows it to be a potent activator of the ATPase of HSP90, with the bulk of its activity residing in the N‐terminal region common to Aha1 and Hch1.^89^ Maximal activation also required the Aha1‐specific C‐terminal domain, but this had no stimulatory activity in isolation.

Subsequent biochemical studies^90^ identified the major binding site for Aha1, as the middle domain of HSP90. Structural studies of the middle domain of yeast HSP90^57^ and its complex with the N‐terminal region of Aha1 91 revealed an extensive interaction between the two that is accompanied by remodelling of the loop bearing the key catalytic residue Arg380. This changes the conformation of the M‐domain catalytic loop towards that seen in the ATP‐bound ‘tense’ state 56 where Arg380 interacts with the γ‐phosphate of the ATP bound to the N‐domain, suggesting that the conserved N‐terminal domain of Aha1 is an allosteric activator of HSP90s ATPase (Figure 8E).

The mechanism of action of the C‐terminal domain of Aha1 – which is required for maximal activation, is less well defined, and no crystal structure incorporating this domain has yet been reported. Heteronuclear NMR studies that confirm the crystallographically‐defined binding site for the N‐terminus of Aha1p on the middle‐domain of HSP90, identified an additional patch of residues on the HSP90 N‐domain that are affected by the binding of full‐length Aha1.^92^ These residues only come into close proximity when the N‐domains of the HSP90 dimer are in the closely associated juxtaposition observed in the ATP‐bound ‘tense’ state of HSP90 56. This suggests that the C‐terminus of Aha1 acts in a similar way to P23/Sba1, stabilizing the transient association of the N‐domains by bridging between protomers. Interestingly a homolog of the C‐terminal domain of Aha1 has been identified in the protozoa *Entamoeba histolytica*, which is able to interact with and stimulate HSP90 in the absence of any equivalent to the Hch1/Aha1‐N domain.^93^

## THE BIOLOGICAL ROLE OF HSP90s ATPase ACTIVITY

The biochemical mechanism by which HSP90 binds and hydrolyses ATP is now very well understood, as are most of the conformational changes that are coupled to that ATPase activity. There is also a reasonable consensus on the mechanisms by which some of the co‐chaperone proteins with which HSP90 interacts, positively or negatively regulate that ATPase, and a growing body of data on how post‐translational modifications may modulate all of this. What still remains very unclear is what this ATPase activity of HSP90 and the conformational chaperone cycle it drives, actually does to or for the wide variety of client proteins that depend on HSP90 for their cellular stability and function.

Early studies attempted to reconcile HSP90 within a protein folding paradigm largely borrowed from studies of the sHSP, CPN60/GroEL and HSP70 families of molecular chaperones, which clearly do play general roles in preventing aggregation and facilitating folding of denatured proteins or proteins trapped in early stages of folding. However elegant genetic studies in yeast showed that HSP90 is not involved in facilitating protein folding as such, but has a focused involvement with selective protein ‘clientele’.^94^ What determines whether a protein is an HSP90 client is also mysterious, although recent studies have suggested that overall thermodynamic stability plays a role.^95^ However such general models are compromised by the intimate involvement of adaptor co‐chaperones in mediating recruitment. Thus selectivity within a client class of which is a ‘client’ and which is not, may depend far more on the affinity for the adaptor than on the nature of the interaction with the chaperone itself. This certainly appears to be the case for HSP90‐dependent protein kinases and their adaptor protein CDC37.^81^

It may be that, despite the weak ‘gearing’ of the ATPase coupled conformational cycle of HSP90,^96^ HSP90 does facilitate conformational rearrangements in the proteins recruited to it, in a comparable way to the *bone fide* ATP‐driven ‘motors’ of the CPN60/GroEL chaperones. This effect could be limited to conformational changes in specific regulatory segments of the clients, such as the ‘activation segment’ in a protein kinase or the C‐terminal helix in the ligand‐binding domain of a steroid hormone receptor, for example. However appealing this idea may be, current structural data on how HSP90 interacts with clients of these types is limited to electron microscopy studies^13^, ^76^, ^78^ whose resolution is still too low to provide the level of detail needed.

An alternative model comes from the widespread observation that, if the ATPase activity of HSP90 is pharmacologically inhibited in a cell, clients such as protein kinases become degraded via the ubiquitin‐dependent proteasome system.^97^, ^98^ At least for proteins kinases, depriving the client of access to the HSP90 system in the first place, by blocking its interaction with CDC37, also promotes their proteasomal degradation.^81^ Taken together these observations suggest that a major function of recruitment to the HSP90 system may be to sequester and stabilize the client until needed, by effectively hiding it from inherent and default degradative pathways. In such a model, the ATPase activity of HSP90 would serve to switch the chaperone conformation between one that alternatively masks and unmasks the client ‘degron’. Further work will be required to distinguish between these functional models.

I gratefully acknowledge the long‐term support of our work on HSP90 by the Wellcome Trust.
